# Immune Checkpoint Inhibitors and Antibody-Drug Conjugates in Urothelial Carcinoma: Current Landscape and Future Directions

**DOI:** 10.3390/cancers17091594

**Published:** 2025-05-07

**Authors:** Shugo Yajima, Hitoshi Masuda

**Affiliations:** National Cancer Center Hospital East, Department of Urology, 6-5-1 Kashiwa no ha, Kashiwa City 277-8577, Japan; hmasuda@east.ncc.go.jp

**Keywords:** urothelial carcinoma, bladder cancer, immunotherapy, immune checkpoint inhibitors, antibody-drug conjugates, pembrolizumab, nivolumab, enfortumab vedotin, combination therapy

## Abstract

Background/Objectives: Urothelial carcinoma (UC) treatment has been transformed by immunotherapy and antibody-drug conjugates (ADCs). This review evaluates the current evidence for these approaches and identifies future directions. Methods: We conducted a structured review of clinical trials, meta-analyses, and guidelines published until early 2025. Results: Immune checkpoint inhibitors have established benefits across multiple settings: post-platinum therapy (pembrolizumab, nivolumab), maintenance therapy (avelumab), adjuvant settings for high-risk muscle-invasive disease (nivolumab), and BCG-unresponsive non-muscle-invasive disease (pembrolizumab). Enfortumab vedotin (targeting Nectin-4) has proven effective in post-platinum/post-immunotherapy. Most significantly, enfortumab vedotin plus pembrolizumab has redefined first-line treatment with unprecedented survival benefits (median OS 31.5 months vs. 16.1 months with chemotherapy; HR 0.47), and nivolumab plus gemcitabine-cisplatin improved outcomes in cisplatin-eligible patients. Key challenges include managing unique toxicity profiles, optimizing treatment sequencing, and developing reliable biomarkers. Conclusions: Combination approaches offer the most promising path forward, with future research needed on resistance mechanisms, biomarker development, and expanding these therapies to earlier disease stages.

## Simple Summary

Urothelial carcinoma (UC) treatment has undergone a revolutionary transformation with the introduction of immunotherapy and antibody-drug conjugates (ADCs). This review examines how immune checkpoint inhibitors (ICIs) have established new standards of care across different stages of UC, from metastatic disease to adjuvant therapy settings. We highlight the emergence of ADCs, particularly enfortumab vedotin, and sacituzumab govitecan, and their impact on clinical practice. Special focus is placed on combination strategies, especially ICI-ADC combinations, which have shown unprecedented survival benefits in first-line settings. Current challenges, including treatment sequencing, resistance mechanisms, and biomarker development, are addressed to provide insights into optimizing patient outcomes.

## 1. Introduction

Urothelial carcinoma (UC) is the most common histological subtype of bladder cancer, with additional occurrences in the upper urinary tract (renal pelvis and ureter) and urethra [1]. UC is broadly classified into non-muscle-invasive bladder cancer (NMIBC), muscle-invasive bladder cancer (MIBC), and locally advanced or metastatic disease (la/mUC) [1]. Prognosis varies significantly by stage, with favorable outcomes in carcinoma in situ (CIS) or localized disease but generally poor prognosis in metastatic settings, where long-term survival rates have historically been quite low [2]. The major risk factors include smoking and occupational exposure to carcinogens [3].

Until recently, the therapeutic options for UC were limited. NMIBC is typically managed with transurethral resection, followed by intravesical therapies such as Bacillus Calmette-Guérin (BCG) [3]. For MIBC, radical cystectomy with neoadjuvant platinum-based chemotherapy is the standard approach [4]. In advanced or metastatic disease, platinum-based chemotherapy regimens, including gemcitabine plus cisplatin (GC) or methotrexate, vinblastine, doxorubicin, and cisplatin (MVAC), have demonstrated modest efficacy, with a median OS of approximately 14–15 months [5]. Unfortunately, approximately 30–50% of patients are ineligible for cisplatin-based therapy due to renal dysfunction or poor performance status [6]. Furthermore, treatment options for patients who progress after platinum-based chemotherapy are historically limited, with poor outcomes [7].

The emergence of immunotherapy, particularly immune checkpoint inhibitors, has revolutionized UC treatment by exploiting the highly immunogenic nature of these tumors [8]. Recently, antibody-drug conjugates (ADCs) have further transformed the treatment landscape by delivering potent cytotoxic agents specifically to tumor cells while minimizing systemic toxicity [9]. The combination of these therapeutic approaches has resulted in unprecedented survival benefits for patients with UC [10].

According to a recent systematic review and meta-analysis, enfortumab vedotin (EV) plus pembrolizumab demonstrated particularly impressive outcomes, with a pooled objective response rate of 68% (95% CI: 64–71%) and a 1-year survival rate of 79% (95% CI: 75–82%) in metastatic UC. These results significantly outperformed chemotherapy in a network meta-analysis, with an odds ratio for 1-year survival of 2.32 (95% CI: 1.75–3.06) [11].

This review provides a comprehensive analysis of the current evidence for immunotherapy and ADCs in UC treatment, examining their mechanisms of action, clinical efficacy, toxicity profiles, and evolving landscape of combination strategies. We also highlight unmet needs, discuss ongoing challenges, and explore future research directionsto further improve outcomes for patients with UC.

## 2. Immune Checkpoint Inhibitors

### 2.1. Mechanism of Action

Immune checkpoint inhibitors function by blocking immune checkpoint pathways that cancer cells exploit to evade immune surveillance. In UC, the primary targets are the programmed cell death protein 1/programmed death-ligand 1 (PD-1/PD-L1) pathway and the cytotoxic T-lymphocyte-associated protein 4 (CTLA-4) pathway [12].

The PD-1/PD-L1 pathway plays a critical role in immune regulation. PD-1, expressed on T cells, interacts with its ligand PD-L1, which can be upregulated on tumor cells and immune cells within the tumor microenvironment. This interaction inhibits T cell activation, proliferation, and cytokine production, thereby suppressing anti-tumor immune responses [13]. Anti-PD-1 antibodies (pembrolizumab, nivolumab) and anti-PD-L1 antibodies (atezolizumab, avelumab, durvalumab) disrupt this interaction, reinvigorating T cell function and anti-tumor immunity [13]. PD-L1 expression levels vary in UC, and their predictive value for treatment response remains controversial [14].

The CTLA-4 pathway is another important immune checkpoint. CTLA-4 is expressed on T cells and competes with CD28 for binding to B7 ligands on antigen-presenting cells, transmitting inhibitory signals that prevent T cell activation. Unlike PD-1/PD-L1, which primarily regulates effector T cell function in peripheral tissues, CTLA-4 modulates T cell activation earlier in the immune response, particularly during priming in the lymphoid organs [15]. Anti-CTLA-4 antibodies (ipilimumab and tremelimumab) block this interaction, enhancing T cell activation. While combination approaches targeting both PD-1/PD-L1 and CTLA-4 pathways have shown promising efficacy in various malignancies, including melanoma and renal cell carcinoma, evidence from studies like CheckMate 032 suggests a similar potential in urothelial carcinoma, where nivolumab plus ipilimumab demonstrated improved response rates compared to monotherapy, particularly with the NIVO1 + IPI3 regimen (1 mg/kg nivolumab plus 3 mg/kg ipilimumab) [16].

### 2.2. Clinical Evidence for ICIs in Different Treatment Settings

#### 2.2.1. Second-Line Treatment After Platinum-Based Chemotherapy

Pembrolizumab established the role of ICIs in UC following the landmark KEYNOTE-045 trial, a randomized phase III study comparing pembrolizumab with the investigator’s choice of chemotherapy (paclitaxel, docetaxel, or vinflunine) in patients with advanced UC who had progressed during or after platinum-based chemotherapy [17]. Pembrolizumab demonstrated significantly improved OS compared to chemotherapy (median 10.3 vs. 7.4 months; hazard ratio [HR] 0.73, *p* = 0.002). This survival benefit was observed regardless of PD-L1 expression level, although it was more pronounced in patients with higher PD-L1 expression (Combined Positive Score [CPS] ≥ 10%; HR 0.57). Notably, progression-free survival (PFS) was not significantly different between the groups. Pembrolizumab showed a more favorable safety profile, with lower rates of treatment-related adverse events [17].

Nivolumab received accelerated approval from the Food and Drug Administration (FDA) for this indication based on the CheckMate 275 phase II trial, which demonstrated an objective response rate (ORR) of 19.6% in patients with platinum-refractory advanced or metastatic UC [18]. Atezolizumab was initially granted accelerated approval based on the phase II IMvigor210 cohort 2 trial. However, the confirmatory phase III IMvigor211 trial failed to demonstrate a significant OS improvement compared to chemotherapy in the primary analysis population of patients with high PD-L1 expression (median OS, 11.1 vs. 10.6 months) [19].

#### 2.2.2. Maintenance Therapy After First-Line Chemotherapy

The JAVELIN Bladder 100 trial established a critical role in the maintenance of immunotherapy in advanced or metastatic UC [20]. This phase III study evaluated avelumab plus best supportive care (BSC) versus BSC alone as maintenance therapy in patients whose disease had not progressed after 4–6 cycles of first-line platinum-based chemotherapy. Avelumab maintenance significantly improved OS compared to BSC alone (median 21.4 vs. 14.3 months; HR 0.69, *p* = 0.001) in the overall population [20]. This study demonstrated that introducing immunotherapy earlier in the disease course, before progression on chemotherapy, could enhance outcomes compared to the historical approach of reserving immunotherapy for disease progression.

#### 2.2.3. First-Line Combination Strategies

The CheckMate 901 trial evaluated nivolumab plus gemcitabine-cisplatin followed by nivolumab maintenance versus GC alone in cisplatin-eligible patients with previously untreated, unresectable, or metastatic UC [21]. This combination demonstrated significantly improved OS (median 21.7 vs. 18.9 months; HR 0.78, *p* = 0.02) and PFS (median 7.9 vs. 7.6 months; HR 0.72, *p* = 0.001) compared with chemotherapy alone. The combination also yielded higher ORR (57.6% vs. 43.1%) and complete response (CR) rates (21.7% vs. 11.8%), with a longer response duration (median CR duration 37.1 vs. 13.2 months) [21].

As discussed in later sections, the most significant advance in first-line therapy has been the combination of enfortumab vedotin with pembrolizumab, which has demonstrated unprecedented survival benefits [10].

#### 2.2.4. Adjuvant Therapy for Muscle-Invasive Urothelial Carcinoma

The CheckMate 274 trial established nivolumab as the standard adjuvant therapy for high-risk muscle-invasive urothelial carcinoma (MIUC) [22]. This phase III trial randomized patients with high-risk MIUC (including both bladder and upper tract primary tumors) who had undergone radical resection to receive either nivolumab or a placebo for up to one year.

Nivolumab significantly improved disease-free survival (DFS) compared to placebo in both the intention-to-treat population (HR 0.70; *p* < 0.001) and patients with tumor PD-L1 expression ≥ 1% (HR 0.55; *p* < 0.001) [22]. In the updated analysis with a longer follow-up (36.1 months), this DFS benefit was maintained (ITT: HR 0.71; PD-L1 ≥ 1%: HR 0.52), and trends toward overall survival improvement emerged (ITT: HR 0.76; PD-L1 ≥ 1%: HR 0.56) [23].

Importantly, the DFS benefit was consistent regardless of whether patients had received prior neoadjuvant chemotherapy [23], providing a valuable option for patients who either did not receive or progressed after neoadjuvant chemotherapy. Based on these results, adjuvant nivolumab has become the standard of care for patients with high-risk MIUC following radical resection.

#### 2.2.5. BCG-Unresponsive Non-Muscle-Invasive Bladder Cancer

Pembrolizumab was approved for BCG-unresponsive, high-risk NMIBC with CIS based on the phase II KEYNOTE-057 trial [24]. In a single-arm study, pembrolizumab demonstrated a complete response rate of 41% in patients with BCG-unresponsive CIS, with a median response duration of 16.2 months [24]. This approval represents an important alternative for patients who would otherwise undergo radical cystectomy after BCG failure.

### 2.3. Management of Immune-Related Adverse Events

Immune checkpoint inhibitors (ICIs) can produce unique toxicity profiles known as immune-related adverse events (irAEs), which result from enhanced immune system activity against normal host tissues [25]. Understanding and managing these toxicities are crucial for optimizing treatment outcomes.

Common irAEs by System

- Dermatologic: Rash, pruritus
- Gastrointestinal: Colitis, diarrhea
- Endocrine: Thyroid dysfunction, hypophysitis, adrenal insufficiency
- Hepatic: Hepatitis
- Pulmonary: Pneumonitis
- Less common but potentially serious: Neurological disorders, hematologic abnormalities, and myocarditis [26]

Management Principles by Severity Grade [27]

Grade 1 (Mild)

- Continue ICI therapy
- Implement symptomatic management
- Monitor symptoms closely
- Patient education on reporting worsening symptoms

Grade 2 (Moderate)

- Temporarily suspend ICI treatment
- Initiate corticosteroids (prednisone 0.5–1 mg/kg/day)
- Consider specialist consultation based on the organ system involved
- Resume ICI once symptoms improve to Grade ≤ 1 and corticosteroid dose ≤ 10 mg/day

Grade 3 (Severe)

- Interrupt ICI treatment
- Administer high-dose corticosteroids (prednisone 1–2 mg/kg/day)
- Consider additional immunosuppressants for steroid-refractory cases
- Hospitalization may be required
- Evaluate the potential permanent discontinuation of ICI therapy

Grade 4 (Life-threatening)

- Permanently discontinue ICI treatment (except for well-controlled endocrinopathies)
- High-dose corticosteroids with hospitalization
- Multidisciplinary management approach
- Consider early additional immunosuppression

Additional Management Considerations

- Taper corticosteroids gradually over 4–6 weeks once symptoms improve
- Consider prophylaxis against opportunistic infections during prolonged corticosteroid use
- Patient education on symptom recognition and prompt reporting is essential
- Several professional organizations, including the American Society of Clinical Oncology and the European Society for Medical Oncology, have published detailed management guidelines for irAEs [27,28]

### 2.4. Biomarkers and Predictors of Response

Despite the clinical benefits of ICIs in UC, reliable predictive biomarkers remain elusive. PD-L1 expression is the most extensively studied biomarker, with various assays and scoring systems employed in clinical trials. Generally, higher PD-L1 expression correlates with improved response rates to ICI therapy. However, responses are also observed in PD-L1-negative tumors, limiting their utility as binary predictive markers [29].

Tumor mutational burden (TMB) is another potential biomarker, with a higher TMB potentially associated with increased neoantigen production and greater immunogenicity [30]. Molecular subtypes of UC (e.g., luminal and basal/squamous), defined through comprehensive genomic analysis, have been investigated for their potential association with immunotherapy response [31]. Other emerging biomarkers include Tumor-Infiltrating Lymphocytes, gene expression signatures reflecting adaptive immune responses or inflammation, and gut microbiome composition [32].

## 3. Antibody-Drug Conjugates

### 3.1. ADC Technology and Principles

Antibody-drug conjugates represent a sophisticated class of targeted therapeutics comprising three key components [33]:

1. Monoclonal antibodies (mAbs): Provide target specificity by binding to antigens preferentially expressed on tumor cells. Ideal target antigens should be abundantly and homogeneously expressed on cancer cells, with minimal expression in normal tissues.
2. Cytotoxic payload: Potent small-molecule drugs that induce cell death and are typically too toxic for conventional systemic delivery. Common payload classes include microtubule inhibitors (e.g., monomethyl auristatin E [MMAE]) and DNA-damaging agents (e.g., SN-38, a topoisomerase I inhibitor).
3. Linker: Chemically connects the antibody to the payload. Linkers must remain stable in circulation while enabling efficient payload release within target cells. Linkers can be designed as cleavable (responding to environmental conditions like low pH or specific enzymes) or non-cleavable (requiring complete antibody degradation).

The mechanism of action begins with antibodies binding to the target antigen on cancer cells, followed by internalization via receptor-mediated endocytosis. Inside the cell, the linker is cleaved (or the antibody is degraded), releasing the payload. The released payload then exerts its cytotoxic effects through mechanisms such as DNA damage or microtubule disruption, ultimately leading to cell death [34].

For ADCs with membrane-permeable payloads and cleavable linkers, the “bystander effect” may occur, wherein released payload molecules diffuse into neighboring tumor cells, potentially killing antigen-negative cells within the heterogeneous tumor microenvironment [35]. Additionally, the antibody component may contribute to therapeutic efficacy through immune-mediated mechanisms, such as antibody-dependent cellular cytotoxicity (ADCC).

### 3.2. Enfortumab Vedotin

#### 3.2.1. Structure and Target

Enfortumab vedotin (EV) consists of a fully humanized IgG1 monoclonal antibody targeting Nectin-4, conjugated to the microtubule-disrupting agent MMAE via a protease-cleavable valine-citrulline linker [36]. Nectin-4 (also known as PVRL4) is a type I transmembrane cell adhesion molecule that belongs to the nectin family. Nectin-4 exhibits limited expression in normal tissues, typically confined to low levels within epithelia. In contrast, it is frequently overexpressed in various malignancies, including urothelial carcinoma (UC). Within UC, Nectin-4 protein expression is particularly high in non-invasive papillary tumors (pTa), reaching 97% positivity [37]. Found in approximately 26% of metastatic UC cases, NECTIN4 amplification predicts markedly enhanced clinical responses and improved survival outcomes with EV therapy [38].

#### 3.2.2. Clinical Evidence

The clinical development of EV began with the EV-101 phase I trial, which showed promising activity (ORR 43%) in patients with previously treated metastatic UC [39]. This led to the pivotal EV-201 trial, a single-arm, phase II study with two cohorts: cohort 1 included patients previously treated with platinum-based chemotherapy and a PD-1/PD-L1 inhibitor, and cohort 2 included cisplatin-ineligible patients who had received prior PD-1/PD-L1 inhibitors [36].

In the EV-201 cohort 1, EV demonstrated an impressive ORR of 44% (including 12% complete responses) and a median duration of response of 7.6 months [36]. Notably, responses were observed across subgroups, including patients with liver metastases and those with prior PD-1/PD-L1 inhibitor resistance. Based on these results, the EV was accelerated for approval.

The confirmatory phase III EV-301 trial randomized patients with locally advanced or metastatic UC who had previously received platinum-based chemotherapy and PD-1/PD-L1 inhibitors to receive either EV or the investigator’s choice of chemotherapy (docetaxel, paclitaxel, or vinflunine) [40]. EV demonstrated significantly improved OS (median 12.9 vs. 9.0 months; HR 0.70, *p* = 0.001) and PFS (median 5.6 vs. 3.7 months; HR 0.62, *p* < 0.001) compared to chemotherapy [40]. These results led to the full approval of EV in this setting.

#### 3.2.3. Safety Profile and Management

The safety profile of enfortumab vedotin (EV) reflects both on-target and off-target effects related to its antibody and payload components [41]. This section organizes the adverse events by severity and provides management recommendations.

Common Adverse Events (Any Grade)

- Skin reactions: Occur in up to 55% of patients [42]
- Peripheral neuropathy: Usually sensory, cumulative with continued treatment
- Fatigue: Common but typically mild to moderate
- Gastrointestinal: Nausea, diarrhea (generally manageable)
- Alopecia: Generally reversible upon treatment discontinuation
- Metabolic: Hyperglycemia (monitor blood glucose, especially in diabetic patients) [11]

Serious Adverse Events (Grade ≥3)

- Severe skin reactions: Grade ≥3 events in approximately 13% of patients, including maculopapular rash, bullous dermatitis, or exfoliative dermatitis [42]
- Severe peripheral neuropathy: Can be dose-limiting
- Ocular disorders: Including conjunctivitis and dry eye [11]
- Severe hyperglycemia: Particularly in patients with pre-existing diabetes

Management Recommendations

Skin toxicity management:

- Early dermatology consultation
- Topical emollients for mild cases
- Topical or systemic corticosteroids for moderate-severe cases
- Consider dose interruption for Grade ≥3 events until resolution to Grade ≤1

Peripheral neuropathy management:

- Regular neurological assessment
- Dose reduction or treatment interruption for Grade ≥2 neuropathy
- Gabapentin or duloxetine may provide symptomatic relief

General management principles:

- Careful baseline and ongoing monitoring
- Prompt intervention at first signs of toxicity
- Appropriate dose modifications according to severity
- Patient education on symptom recognition and reporting

Despite the high incidence of adverse events, most are manageable with proper supportive care, allowing the continuation of treatment in many patients [41,42].

### 3.3. Sacituzumab Govitecan

#### 3.3.1. Structure and Target

Sacituzumab govitecan (SG) is composed of a humanized IgG1κ monoclonal antibody targeting Trophoblast cell-surface antigen 2 (Trop-2) conjugated to SN-38, the active metabolite of irinotecan and a potent topoisomerase I inhibitor [43]. The linker is a hydrolyzable CL2A linker that allows for both intracellular and extracellular release of SN-38. SG has a high drug-to-antibody ratio of approximately 7.6, delivering a higher payload than that of many other ADCs [44].

Trop-2 (also known as TACSTD2) is a transmembrane glycoprotein involved in calcium signaling, cell proliferation, and migration [45]. While its expression can be heterogeneous, Trop-2 is frequently and highly expressed in UC [46]. An analysis of archival tumor samples from 146 patients enrolled in cohorts 1–3 of the TROPHY-U-01 trial confirmed this high prevalence; using immunohistochemistry (IHC), Trop-2 expression (defined as H-score > 0) was detected in 98% of evaluable samples, with a median H-score of 215 (on a 0–300 scale) [46]. Consistent with this widespread expression, further analyses within the TROPHY-U-01 study demonstrated that the efficacy of SG (including ORR, PFS, and OS) was observed across the spectrum of Trop-2 IHC expression levels, suggesting that clinical benefit is not strictly dependent on the measured level of Trop-2 expression [43].

#### 3.3.2. Clinical Evidence

The TROPHY-U-01 trial, a phase II open-label study, evaluated the efficacy of SG in metastatic UC across multiple cohorts [43]. Cohort 1 included patients who were previously treated with platinum-based chemotherapy and PD-1/PD-L1 inhibitors. SG demonstrated an ORR of 27%, a median duration of response of 7.2 months, and a median OS of 10.9 months [43]. This led to the accelerated approval of SG for this indication.

Cohort 2 evaluated SG in cisplatin-ineligible patients previously treated with PD-1/PD-L1 inhibitors, showing an ORR of 32%, a median duration of response of 5.6 months, and a median OS of 13.5 months [47].

The confirmatory phase III TROPiCS-04 trial compared SG to the investigator’s choice of chemotherapy in patients with advanced UC who had progressed after platinum-based chemotherapy and PD-1/PD-L1 inhibitors [48]. Unfortunately, SG did not demonstrate statistically significant improvements in the primary endpoint of OS (median 10.3 vs. 9.0 months; HR 0.86, *p* = 0.087) or PFS (median 4.2 vs. 3.6 months; HR 0.86) [48]. The ORR was higher with SG (23% vs. 14%); however, this did not translate into survival benefits. These results raise questions regarding the role of SG in UC treatment.

#### 3.3.3. Safety Profile and Management

The toxicity profile of SG primarily reflects its payload, SN-38, and includes hematologic abnormalities (neutropenia, anemia, and leukopenia) and gastrointestinal symptoms (diarrhea, nausea, and vomiting) [49].

Neutropenia is the most significant toxicity, occurring in up to 64% of patients, including febrile neutropenia in approximately 7% of patients [49]. Management typically involves granulocyte colony-stimulating factor support, dose reduction, and treatment delay. Close monitoring, particularly during the early treatment cycles, is essential due to the risk of neutropenic complications.

Diarrhea affects approximately 62% of patients (Grade ≥ 3 in 8%) and may be managed with loperamide, atropine for cholinergic symptoms, and dose adjustments [50]. Other common adverse events include nausea, vomiting, fatigue, and alopecia, which are generally manageable with standard supportive care.

### 3.4. Other ADCs in Development

Several other ADCs targeting different antigens are being developed for UC, including:

HER2-targeted ADCs: Human epidermal growth factor receptor 2 (HER2) is a therapeutic target in UC. A systematic literature review found that HER2 positivity occurs in approximately 13.0% of patients with locally advanced or metastatic UC [51]. Disitamab vedotin, an anti-HER2 ADC, has demonstrated significant activity in this population. In a combined analysis of two phase II trials involving patients with HER2-positive (defined as IHC 2+ or 3+) locally advanced or metastatic UC who had progressed on prior systemic chemotherapy, disitamab vedotin achieved a confirmed ORR of 50.5% (95% CI, 40.6% to 60.3%) as assessed by a blinded independent review committee [52].

Novel Nectin-4-targeted ADCs: Next-generation ADCs targeting Nectin-4 are being developed with modified linkers or conjugation technologies to potentially improve tolerability. For example, CRB-701 aims to reduce peripheral neuropathy and skin reactions compared to EV through technology refinements [53].

## 4. Combination Strategies: ICIs and ADCs

### 4.1. Rationale for Combination Approaches

The combination of ICIs and ADCs represents a promising therapeutic strategy that enhances anti-tumor efficacy through complementary mechanisms of action. This approach is supported by several biological principles.

First, ADCs induce immunogenic cell death (ICD), characterized by the release of tumor-associated antigens and damage-associated molecular patterns (DAMPs), which stimulate immune recognition [54]. This process can potentially convert immunologically “cold” tumors with minimal immune cell infiltration into “hot” tumors with enhanced immune responsiveness, potentially increasing their sensitivity to ICI therapy. Second, ICIs can amplify and sustain the anti-tumor immune responses initiated by ADC-induced tumor cell death, potentially extending the duration of the clinical response [55]. Furthermore, combining agents with distinct mechanisms of action may effectively overcome or delay the development of drug resistance pathways that frequently limit the efficacy of monotherapy [56].

The clinical validation of these principles is evident in studies comparing combination and monotherapy approaches. For instance, the EV-103 trial demonstrated that the combination of EV with pembrolizumab achieved significantly higher confirmed objective response rates than EV monotherapy (64.5% versus 45.2%) in cisplatin-ineligible patients with previously untreated locally advanced or metastatic UC [57]. Through the interplay of these mechanisms, ICI-ADC combinations demonstrate synergistic therapeutic effects that exceed those achievable with either agent alone.

### 4.2. Enfortumab Vedotin Plus Pembrolizumab

The combination of EV and the immune checkpoint inhibitor pembrolizumab has emerged as a groundbreaking and highly effective treatment strategy for advanced UC.

The initial evaluation of this combination was conducted in the multi-cohort phase Ib/II EV-103 trial [57]. Cohort K of this study directly compared EV plus pembrolizumab with EV monotherapy in cisplatin-ineligible patients with previously untreated locally advanced or metastatic UC. This comparison favored the combination, demonstrating a higher confirmed ORR (64.5% vs. 45.2%) [57]. A meta-analysis of early phase results reported a pooled ORR of 68% (95% CI, 64–71%) and a DCR of 86% (95% CI, 83–89%) for the combination in the first-line setting [11].

The pivotal phase III EV-302/KEYNOTE-A39 trial provided definitive evidence supporting this combination therapy [10]. This landmark study compared EV plus pembrolizumab with standard platinum-based chemotherapy in patients with previously untreated locally advanced or metastatic UC, encompassing both cisplatin-eligible and-ineligible individuals [10]. The trial met its primary endpoints, demonstrating remarkable improvements with combination therapy. The median OS was significantly prolonged (31.5 months vs. 16.1 months; HR 0.47, *p* < 0.001), as was the median PFS (12.5 months vs. 6.3 months; HR 0.45, *p* < 0.001) compared to chemotherapy [10]. The ORR was also significantly higher with EV plus pembrolizumab (67.7% vs. 44.4%, *p* < 0.001) [10]. These outcomes represent a substantial improvement over the historical results of chemotherapy or immunotherapy alone [10].

In addition to efficacy advantages, the EV-pembrolizumab combination also offers a more favorable hematologic toxicity profile than standard chemotherapy. According to a meta-analysis, this combination was associated with significantly lower rates of all-grade anemia than chemotherapy (OR, 0.20; 95% CI, 0.05–0.84; *p* = 0.03) [11]. While not statistically significant, both EV-based regimens demonstrated numerically lower rates of neutropenia than chemotherapy [11]. This reduced hematologic toxicity represents a meaningful clinical advantage, particularly for elderly patients and those with comorbidities who may be more vulnerable to developing myelosuppression.

### 4.3. Sacituzumab Govitecan Plus Pembrolizumab

The combination of SG and pembrolizumab has been evaluated in the TROPHY-U-01 cohort 3. This cohort enrolled patients with mUC who had progressed after platinum-based chemotherapy but were naive to checkpoint inhibitors [58]. This phase II study demonstrated promising efficacy, with an ORR of 41% (95% CI, 26.3–57.9) per central review, including a CR rate of 20% [58]. The median duration of response was 11.1 months, median PFS was 5.3 months, and the median OS was 12.7 months [58]. The safety profiles were consistent with the known toxicities of each agent. Grade ≥ 3 treatment-related AEs occurred in 61% of patients, with the most common being neutropenia (37%), leukopenia (20%), and diarrhea (20%) [58]. No unexpected safety signals were observed [58].

While these results are encouraging, the clinical positioning of this combination remains uncertain, particularly given the emergence of EV plus pembrolizumab [10] and nivolumab plus GC [21] as first-line treatments. Further evaluation in randomized trials is needed to establish its role in the evolving treatment landscape.

## 5. Conclusions

**Figure 1:**
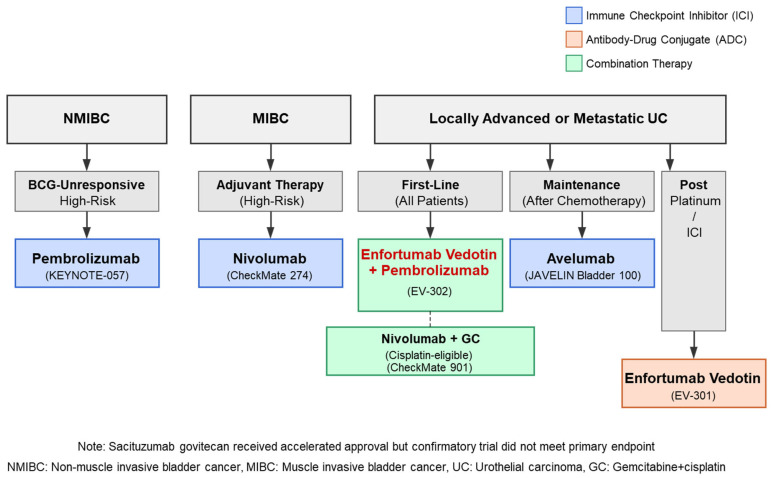
Treatment Algorithm for Urothelial Carcinoma by Disease Stage. Standard treatment approaches for urothelial carcinoma by disease stage based on pivotal clinical trials in 2025. Color coding: blue (immune checkpoint inhibitors), orange (antibody-drug conjugates), and green (combination therapies). The first-line preference for enfortumab vedotin plus pembrolizumab in metastatic disease represents a major paradigm shift in the treatment.

Immunotherapy and antibody-drug conjugates have fundamentally transformed urothelial carcinoma treatment. As illustrated in Figure 1 and Table 1, immune checkpoint inhibitors have established important roles across multiple disease settings: pembrolizumab and nivolumab after platinum-based chemotherapy, avelumab as maintenance therapy, nivolumab in the adjuvant setting for high-risk muscle-invasive disease, and pembrolizumab for BCG-unresponsive non-muscle-invasive disease. Enfortumab vedotin has demonstrated significant efficacy in advanced disease after platinum and immunotherapy failure.

The paradigm shift in UC treatment has been primarily driven by combination approaches. EV plus pembrolizumab has delivered unprecedented survival benefits in first-line metastatic treatment (median OS 31.5 vs. 16.1 months; HR 0.47) [10], while nivolumab plus gemcitabine-cisplatin has improved outcomes for cisplatin-eligible patients (median OS 21.7 vs. 18.9 months; HR 0.78) [21]. These advances have rapidly altered standard therapeutic pathways, creating a framework for precision medicine across all disease stages.

The mechanisms of resistance to both ICIs and ADCs represent a critical area for further investigation. Primary and acquired resistance to ICIs may involve alterations in the antigen presentation machinery, T-cell exclusion, immunosuppressive microenvironments, and activation of alternative immune checkpoints. Resistance to ADCs can develop through the downregulation of target antigens, alterations in internalization mechanisms, enhanced drug efflux, and changes in apoptotic pathways. Further elucidation of these mechanisms will guide the rational development of next-generation combination strategies to achieve more durable disease control.

Looking ahead, four key priorities will define the evolution of UC treatment: (1) development of biomarkers to optimize patient selection, (2) novel ADC targets beyond Nectin-4 and Trop-2, (3) innovative combination strategies incorporating other therapeutic modalities, and (4) expansion of these approaches into earlier disease settings. As translational research advances our understanding of resistance mechanisms and treatment response determinants, we are moving closer to the goal of long-term disease control for patients with this challenging malignancy.

## Figures and Tables

**Table 1:** Summary of Key Clinical Trials of ICIs and ADCs in Urothelial Carcinoma.

Trial	Phase	Population	Treatment	Primary Endpoint(s)	Key Results	Grade ≥ 3 AEs	Reference
KEYNOTE-045	III	Post-platinum	Pembrolizumab vs. Chemo	OS	10.3 vs. 7.4 mo (HR 0.73)	15% vs. 49%	[17]
IMvigor211	III	Post-platinum	Atezolizumab vs. Chemo	OS	11.1 vs. 10.6 mo (NS)	20% vs. 43%	[19]
JAVELIN Bladder 100	III	Maintenance after first-line	Avelumab + BSC vs. BSC	OS	21.4 vs. 14.3 mo (HR 0.69)	16.6% vs. 0%	[20]
CheckMate 274	III	Adjuvant	Nivolumab vs. Placebo	DFS	HR 0.70, *p* < 0.001	17.9% vs. 7.2%	[22,23]
EV-301	III	Post-platinum/post-ICI	EV vs. Chemo	OS	12.9 vs. 9.0 mo (HR 0.70)	51.4% vs. 49.8%	[40]
EV-302/KEYNOTE-A39	III	First-line	EV + pembro vs. Chemo	OS, PFS	OS: 31.5 vs. 16.1 mo (HR 0.47)	55.9% vs. 69.5%	[10]
CheckMate 901	III	First-line (cisplatin-eligible)	Nivo + GC vs. GC	OS, PFS	OS: 21.7 vs. 18.9 mo (HR 0.78)	61.8% vs. 51.7%	[21]
TROPiCS-04	III	Post-platinum/post-ICI	SG vs. Chemo	OS	10.3 vs. 9.0 mo (HR 0.86, NS)	67% vs. 35%	[48]

Abbreviations: AEs, adverse events; BSC, best supportive care; Chemo, chemotherapy; DFS, disease-free survival; EV, enfortumab vedotin; GC, gemcitabine + cisplatin; HR, hazard ratio; ICI, immune checkpoint inhibitor; mo, months; Nivo, nivolumab; NS, not significant; OS, overall survival; pembro, pembrolizumab; PFS, progression-free survival; SG, sacituzumab govitecan.

## Abbreviations

NMIBC: Non-muscle-invasive bladder cancer, MIBC: Muscle-invasive bladder cancer, UC: Urothelial carcinoma, GC: Gemcitabine + cisplatin.
